# Relationship Between Tendon Tissue and Shoulder Disability Change During an 8-Week Exercise Intervention for Rotator Cuff Tendinopathy: An Observational Study

**DOI:** 10.1093/ptj/pzaf107

**Published:** 2025-08-29

**Authors:** Oscar Vila-Dieguez, Matt D Heindel, Mark C Zipser, Kameron Mortazavi, Kornelia Kulig, Greg Bashford, Wendy Mack, Lori A Michener

**Affiliations:** Division of Biokinesiology and Physical Therapy, University of Southern California, Los Angeles, CA 90089, United States; Division of Biokinesiology and Physical Therapy, University of Southern California, Los Angeles, CA 90089, United States; Division of Biokinesiology and Physical Therapy, University of Southern California, Los Angeles, CA 90089, United States; Division of Biokinesiology and Physical Therapy, University of Southern California, Los Angeles, CA 90089, United States; Division of Biokinesiology and Physical Therapy, University of Southern California, Los Angeles, CA 90089, United States; Department of Biological Systems Engineering, University of Nebraska–Lincoln, Lincoln, NE 68588, United States; Department of Population and Public Health Sciences, University of Southern California, Los Angeles, CA 90089, United States; Division of Biokinesiology and Physical Therapy, University of Southern California, Los Angeles, CA 90089, United States

**Keywords:** Mechanisms, Patient-reported Outcomes, Resistive Exercise, Tendon Morphology

## Abstract

**Importance:**

Understanding how tendon structure relates to disability improvement during exercise interventions in rotator cuff tendinopathy is essential for optimizing individualized treatment strategies.

**Objective:**

The objectives of this study were to characterize changes in supraspinatus tendon thickness and internal architecture over an 8-week resistive exercise intervention and evaluate the relationship between these changes and patient-reported shoulder disability.

**Design:**

This was a prospective longitudinal observational study.

**Setting:**

The settings were a university-based research laboratory and virtual supervision in participants’ homes.

**Participants:**

Forty-seven adults with unilateral rotator cuff tendinopathy were the study participants.

**Intervention:**

The intervention was an 8-week progressive resistive exercise program, supervised twice weekly by a physical therapist.

**Main Outcomes and Measures:**

Primary outcomes were the Pennsylvania Shoulder Score, supraspinatus tendon thickness, and internal tendon architecture assessed using the peak spatial frequency radius via ultrasound. Measurements were taken at baseline and at 2, 4, and 8 weeks. Linear mixed-effects models were used to assess changes and associations.

**Results:**

Significant improvements from baseline were observed for the Pennsylvania Shoulder Score at 2, 4, and 8 weeks. Tendon thickness decreased significantly; changes in internal tendon architecture were not significant. A decrease in tendon thickness was associated with an improved Pennsylvania Shoulder Score at 2 weeks but not at 4 and 8 weeks.

**Conclusions:**

Reductions in tendon thickness were associated with improved shoulder outcomes within the first 2 weeks of the intervention. Internal tendon architecture remained unchanged over the intervention. These findings suggest that tendon variables included in this study may be relevant only early in the intervention and that other factors should be investigated across different times of the intervention.

## INTRODUCTION

Rotator cuff tendinopathy is the most common cause of musculoskeletal shoulder pain,^1^^,^^2^ and the leading contributor to shoulder-related disability in adults.^2^^,^^3^ Physical therapy is the first-line treatment for rotator cuff tendinopathy, and resistive exercise is recommended in all clinical practice guidelines.^4^^,^^5^ Resistive exercise is beneficial in improving shoulder disability,^6–9^ but 40% to 50% of patients develop recurrent or chronic symptoms.^10–13^ Clinical trials comparing resistive exercise to other interventions such as education or corticosteroid injection find similar group-level changes in patient-reported outcomes, despite these treatments being theorized to work via different mechanisms.^14–16^ This highlights the need to understand the mechanisms that explain patient response to intervention.

One potential mechanism is the change in tendon morphology.^17^ Patients with rotator cuff tendinopathy often present with deficits in supraspinatus tendon morphology.^18–28^ Regarding macromorphology or tendon size, cross-sectional studies report the tendon is thicker in those with rotator cuff tendinopathy compared to controls who are healthy.^19^^,^^22^^,^^24–26^ This is attributed primarily to increased water content, neovascularization, and proinflammatory cells.^29–34^ However, longitudinal studies investigating changes in tendon morphology after intervention are limited.^35–37^ These studies have shown contradicting results. Two studies examining the injured shoulder only, found no change in tendon thickness over the course of a resistive exercise intervention.^35^^,^^36^ A large trial using a bilateral ratio found increased thickness in those with thinner tendons and decreased thickness in those with thicker tendons.^37^ This study used bilateral tendon size difference as the variable of interest over the course of an intervention.^37^ However, this assumes that the noninvolved side remains unchanged. Given the systemic nature of tendinopathy, assessing the local tendon size change in addition to the bilateral difference is warranted. Another gap in prior studies is the lack of quantification of the association between changes in tendon morphology and patient-reported disability. Achilles tendinopathy research has found patient subgroups with minimal tendon morphology deficits.^38^ This challenges the expectation of uniform group-level tendon morphology changes and supports patient-specific exploration of the relationship with patient-reported disability.

Another gap in the literature is the characterization of tendon micromorphology or internal tendon architecture in rotator cuff tendinopathy. Water content comprises ~55% of the weight of healthy tendons,^32^^,^^33^ but the majority of a tendon’s dry weight is collagen.^32^^,^^33^ During initial tendon tissue repair, collagen appears in an irregular disorganized alignment,^34^^,^^39^ impacting tendon architecture and mechanical properties.^40^^,^^41^ One method to quantify tendon internal architecture is spatial frequency analysis of a gray-scale ultrasound image.^40^^,^^42–44^ Only 2 cross-sectional studies have used this method for the supraspinatus tendon: 1 of them found altered internal tendon architecture in swimmers with higher pain levels ^45^, whereas the other found no difference between individuals with and those without rotator cuff tendinopathy.^46^ No longitudinal studies have characterized internal tendon architecture changes after resistive exercise, so it is unknown if these relate to patient response to resistive exercise.

The purpose of this study is to characterize the changes in tendon morphology and patient-reported outcomes and the relationship between these changes over time in patients with rotator cuff tendinopathy.

## METHODS

Participants (*N* = 47) with unilateral rotator cuff tendinopathy were recruited via flyers at surrounding clinics and athletic facilities, and via solicitation through an internal patient directory of the research institution. Inclusion criteria were age of 18 to 55 years (those >55 years old were excluded because of an increased likelihood of a full-thickness tendon tear^47^), Pennsylvania Shoulder Score (Penn Score) of ≤85/100 (100 = no disability) to allow for a minimal detectable change (MDC) of 12.1 points,^48^ a diagnosis of rotator cuff tendinopathy via 3 of 5 positive clinical tests,^49–51^ and a minimum duration of symptoms of 3 months to avoid acute transient pain. Exclusion criteria included the presence of neck/thoracic pain or shoulder pain reproduced with Spurling, cervical rotation, or axial compression tests^52^; prior surgery on the shoulder, neck, or thoracic spine; primary adhesive capsulitis (passive range of motion loss of >50% in external rotation or elevation)^51^^,^^53^; instability indicated by positive anterior or posterior apprehension tests^51^^,^^53^; and full-thickness rotator cuff tear, verified by ultrasound imaging.^47^ Partial-thickness tears were included given the similarity of clinical presentation and prognosis with conservative treatment compared to tendinopathy without tears. Informed consent was obtained from all participants prior to their enrollment in the study, and all procedures were conducted in accordance with the ethical standards of the Declaration of Helsinki. This study was approved by the Institutional Review Board of the University of Southern California (approval number: HS-20-00994).

### Procedures

#### Resistance Exercise Program

The standardized resistance exercise program used previously in 2 clinical trials.^54^^,^^55^ The program comprised of 3 phases of motor control and strengthening exercises. Pain, effort and ability to complete the prescribed number of sets and repetitions were monitored for progression, which makes the exercise intervention standardized while accounting for different physical activity levels. Full progression algorithm is included in Supplementary Material 1.^55^ On the basis of previous literature reporting significant changes in patient-reported outcomes within 6 to 8 weeks, 8 weeks was selected for the duration of the intervention.^7^ Participants performed the exercises 5 times per week. Two exercise sessions per week were supervised by a licensed physical therapist in person or virtually.

#### Patient-Reported Outcomes

The Penn Score consists of 3 subscales: pain, function and satisfaction. The total score is 0 to 100 (where 100 = no shoulder disability). The Penn Score has demonstrated reliability, validity, and responsiveness in patients with shoulder pain.^48^^,^^56^ The average MDC is 12.1 points, and the minimum clinically important difference is 11.4 points.^48^

#### Tendon Morphology

**Figure 1:**
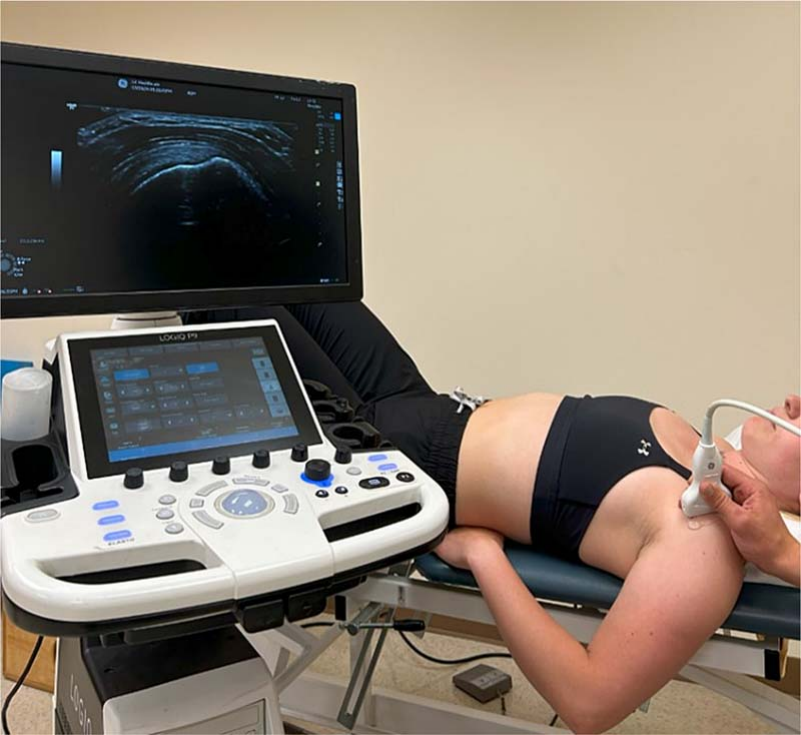
Longitudinal Image Acquisition of the Supraspinatus Tendon for Thickness Measurement.

Ultrasound images of the supraspinatus tendon were obtained with a 6- to 12-MHz linear transducer (P9; GE Health care, Wauwatosa, WI, USA). Gray-scale B-mode images were obtained with the participant in a supine position and with the arm over the edge of the table in the Crass position to expose the tendon (Figure 1). The transducer was placed on the anterior aspect of the shoulder, parallel to the tendon. The supraspinatus was the focus because it is the most commonly affected tendon in rotator cuff tendinopathy.^18–28^

Tendon thickness was measured at 5, 10, and 15 mm proximal to the tendon attachment to the greater tuberosity in the longitudinal view. The 3 measurements were averaged to represent tendon thickness.^22^^,^^46^ Test–retest reliability performed with 10 participants (ICC = 0.97; MDC = 0.3 mm) was similar to that in prior studies.^22^^,^^57^^,^^58^

**Figure 2:**
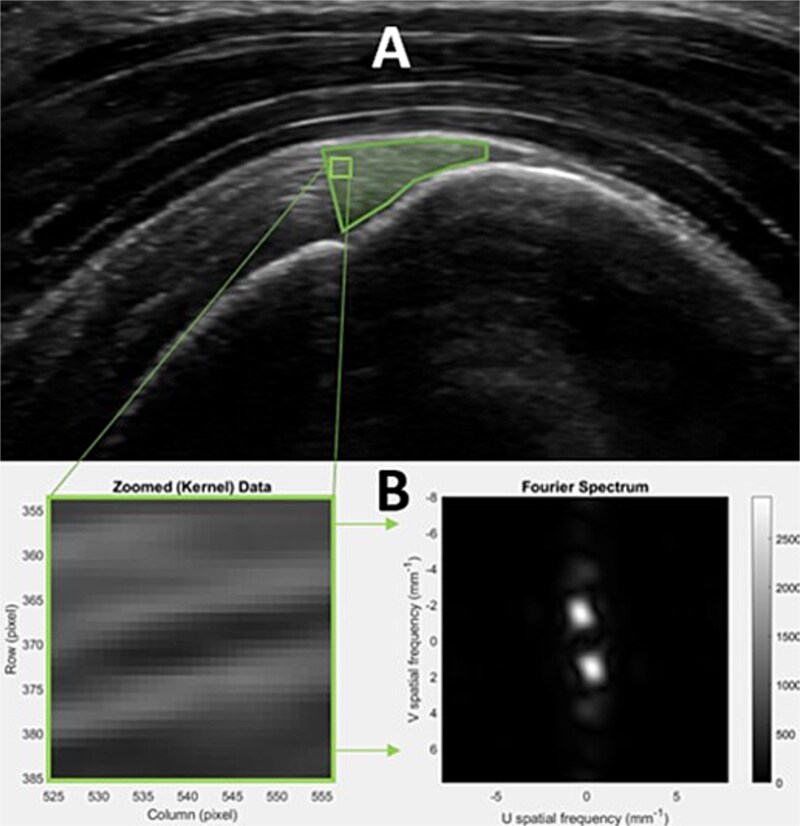
Data Analysis Procedure for Extraction of Spatial Frequency Analysis (SFA) Parameters From Images. (A) Region-of-interest drawing and kernel example. (B) Zoomed kernel and fast Fourier transformation process for SFA parameter extraction. Abbreviations: U = spatial frequency in the horizontal direction; V = spatial frequency in the vertical direction.

Internal tendon architecture was assessed using spatial frequency analysis, an image analysis method based on the spatial spectra of sub-images (“kernels”) of an image.^44^ A region of interest within the longitudinal image was selected first (Figure 2). The region of interest for the spatial frequency analysis was drawn from the anatomical neck of the humerus superiorly to the border of the bursa, following it distally to the apex of the greater tuberosity, and following the lower border of the tendon medially back to the anatomical neck. All images were processed using previously described custom MATLAB algorithms (The MathWorks, Inc, Natick, MA, USA) and extraction of spatial frequency analysis parameters.^44^ The peak spatial frequency radius (PSFR) parameter was used to characterize internal tendon architecture. The PSFR is the distance between the origin and the spatial frequency peak in the 2-dimensional spatial spectrum and represents the dominant spacing of tissue fascicles/fibers. Higher PSFR values indicate more organization of the collagen fibers.^40^^,^^59^ Test–retest reliability was established for this measure with 10 participants (ICC = 0.75; MDC = 0.08 mm^−1^).

### Sample Size

Given the lack of longitudinal studies of changes in tendon morphology, a 2-week pilot study with 10 participants was conducted to inform the sample size calculation for the study with 4 time points; variance components were estimated from these data for the sample size estimation for the main study. The “longpower” package in R (R Foundation for Statistical Computing, Vienna, Austria) was used (power = 0.8; Cohen d = 0.5; α = .05). Analysis indicated that 33 participants were needed, and accounting for a 20% dropout over 8 weeks, we aimed to recruit a minimum of 41 participants.

### Data Analysis

We employed linear mixed-effects models to examine the relationship between longitudinal changes in tendon morphology (tendon thickness and PSFR) as independent variables and patient-reported outcomes (Penn Score) as the dependent variable. These models included a participant-level random intercept to account for the repeated-measures design and variability between participants. All statistical analyses were conducted using R (v4.0.0).

#### Did Patient-Reported Outcomes and Tendon Structure Change Over the 8-Week Intervention?

The first 3 models assessed whether the Penn Score, tendon thickness, and PSFR (dependent outcome variables) changed significantly as a function of weeks (factor). The model equation for each outcome was as follows:


$$ {Y}_{it}={\mathrm{\beta}}_0+{\mathrm{\beta}}_1\,{\mathrm{week}}_2+{\mathrm{\beta}}_2\,{\mathrm{week}}_4+{\mathrm{\beta}}_3\,{\mathrm{week}}_8+{u}_i+{\mathrm{\varepsilon}}_{i\mathrm{t}}, $$

where ${Y}_{it}$ is the outcome for participant $i$ at time $t$; ${\mathrm{\beta}}_0$ is the intercept, representing the baseline level of the outcome; $\beta$_1_ week_2_, $\beta$_2_ week_4_, and $\beta$_3_ week_8_ are fixed effects representing changes at weeks 2, 4, and 8, respectively, relative to the baseline; and ${u}_i$ and ${\mathrm{\varepsilon}}_{it}$ represent the random intercept and residual error, respectively.

#### Were Changes in Tendon Structure Associated With Changes in Patient Outcomes?

Two models addressed this question. One model assessed bilateral tendon structure (calculated as the difference between sides), and 1 assessed the involved shoulder only. To capture all possible relationships, the model was run first with the changes calculated from baseline. Then, the model was run again with changes calculated sequentially: baseline to week 2, week 2 to week 4, and week 4 to week 8. The first model assessed change in Penn Score (dependent outcome variable) as a function of weeks (factor), change in bilateral tendon thickness difference (involved side minus noninvolved side), and change in bilateral PSFR difference. Hand dominance, time since onset of pain, baseline weight, baseline tendon thickness difference, baseline PSFR, and baseline Penn Score were included as covariates:


\begin{align*} &{\Delta \mathrm{Penn}}_{it}={\mathrm{\theta}}_0+\sum_j{\mathrm{\lambda}}_{\mathrm{j}}{\mathrm{covariate}}_{ij}+{\mathrm{\beta}}_1\left({\mathrm{week}}_2\times \Delta \mathrm{TT}\right)\\&\qquad+{\mathrm{\beta}}_2\left({\mathrm{week}}_4\times \Delta \mathrm{TT}\right)+{\mathrm{\beta}}_3\left({\mathrm{week}}_8\times \Delta \mathrm{TT}\right) \\&\qquad+{\mathrm{\gamma}}_1\left({\mathrm{week}}_2\times \Delta \mathrm{PSFR}\right)+{\mathrm{\gamma}}_2\left({\mathrm{week}}_4\times \Delta \mathrm{PSFR}\right)\\&\qquad+{\mathrm{\gamma}}_3\left({\mathrm{week}}_8\times \Delta \mathrm{PSFR}\right)+{u}_i+{\mathrm{\varepsilon}}_{\mathrm{it}},\end{align*}

where ${\Delta \mathrm{Penn}}_{it}$ is the Penn Score change for participant $i$ at time $t$; ${\mathrm{\theta}}_0$ is the intercept, representing the first level of Penn Score change; ${\mathrm{\lambda}}_{\mathrm{j}}$ represents the coefficient for covariates; $\beta$_1_, $\beta$_2_, and $\beta$_3_ represent interactions for tendon thickness change; $\mathrm{\gamma}$_1_, $\mathrm{\gamma}$_2_, and $\mathrm{\gamma}$_3_ represent interactions for PSFR change; and ${u}_i$ and ${\mathrm{\varepsilon}}_{\mathrm{it}}$ represent the random intercept and residual error, respectively.

The second model focused on the involved shoulder, examining the change in Penn Score as a function of weeks, and the interaction of weeks with changes in tendon thickness and PSFR. Same covariates from the previous model were included.

Multicollinearity was assessed using variance inflation factors for each model. Leave-one-out cross-validation was performed to assess predictive accuracy of the models. The root-mean-square error (RMSE) was calculated to evaluate model performance across all folds.

## RESULTS

Table 1 summarizes participant demographics. From the 47 participants, 40 completed all visits and 7 participants missed 1 of the 4 in-person visits because of scheduling conflicts or personal reasons. Patient-reported outcomes were still collected online, but tendon measures were missing data for that visit. Linear mixed-effects models allowed for robust estimation using maximum likelihood techniques without biasing results when the data was missing at random. Adherence to exercise program was high, with a low number of missed sessions reported (3 [SD = 3.3] of 40 total sessions).

**Table 1:** Participant Demographics*^a^*

Characteristic	Value
Time since onset of pain, mo, median (IQR)	6 (18)
Height, cm, mean (SD)	169 (10)
Weight, kg, mean (SD)	72 (16)
BMI, mean (SD)	24.98 (4.83)
Age, y, mean (SD)	31 (10)
Comorbidity	13 (27.7)
Obesity	7 (14.9)
Smoking	7 (14.9)
High cholesterol	6 (12.7)
Hypertension	3 (6.4)
Diabetes	1 (2.1)
Psoriasis	2 (4.2)
Dominant side	
Right	41 (87.2)
Left	3 (6.4)
Ambidextrous	3 (6.4)
Painful side	
Dominant	32 (68.1)
Nondominant	15 (31.9)
Ethnicity	
Hispanic or Latino	4 (8.5)
Not Hispanic or Latino	43 (91.5)
Race	
White or Caucasian	24 (51.1)
Asian	12 (25.5)
Multiple races	6 (12.8)
Other	5 (10.6)
Sex	
Male	32 (68.1)
Female	14 (29.8)
Prefer not to answer	1 (2.1)

^*a*^Data are presented as numbers (percentages) of participants unless otherwise indicated. Normally distributed data are presented as mean (SD). Nonnormally distributed data are presented as median (interquartile range [IQR]). BMI = body mass index.

### Did Patient-Reported Outcomes and Tendon Structure Change Over the 8-Week Intervention?

The linear mixed-effects model assessing the change in Penn Score by study of weeks revealed significant improvements at week 2 (mean = 6.3; SE = 1.2; *P* < .001), week 4 (mean = 11.2; SE = 1.2; *P* < .001), and week 8 (mean = 17.4; SE = 1.2; *P* < .001) compared to baseline, only reaching the minimum clinically important difference at week 8 for the Penn Score. The linear mixed-effects model assessing the change in tendon thickness over time revealed significant reductions at week 2 (mean = −0.09; SE = 0.03; *P* < .001), week 4 (mean = −0.16; SE = 0.03; *P* < .001), and week 8 (mean = −0.20; SE = .03; *P* < .001), but they were smaller than our MDC (0.3 mm). The PSFR did not change significantly at any time point.

### Were Changes in Tendon Structure Associated With Changes in Patient Outcomes?

The linear mixed-effects models assessing changes in the Penn Score as a function of weeks and the interaction of weeks with changes in bilateral tendon thickness difference and PSFR difference did not reveal significant interactions or main effects for these variables. The covariate baseline Penn Score was the only significant variable (estimate = −0.2; SE = 0.08; *P* = .004), indicating that lower baseline scores were associated with more improvement in the Penn Score.

The linear mixed-effects models assessing changes in the Penn Score as a function of weeks and the interaction of weeks with changes in tendon thickness and PSFR on the involved side (Table 2) revealed that the change in tendon thickness was associated with a change in the Penn Score at week 2. Specifically, for every 1-mm decrease in tendon thickness, there was a 14.4-point increase in the Penn Score, with an RMSE of 5.6 points on the basis of the leave-one-out cross-validation for this model. Tendon thickness and PSFR changes were not significantly related to the Penn Score at weeks 4 and 8. The baseline Penn Score was also significantly associated with the change in the Penn Score. This model explained a small amount of variance in Penn Score changes, with an R^2^ value of 0.17. Complete outputs for all models are included in Supplementary Material 2.

**Table 2:** Estimates for the Model With Changes Calculated Sequentially*^a^*

Parameter	Estimate	SE	t	P
Intercept	11.90	10.10	1.18	.24
Baseline tendon thickness	0.56	0.88	0.63	.53
Baseline PSFR	−0.28	4.12	−0.07	.95
Pain in dominant vs nondominant side	0.39	1.18	0.33	.74
Time since onset of pain	−0.00	0.00	−0.55	.59
Baseline weight	0.02	0.02	1.14	.26
Baseline Pennsylvania shoulder score	−0.17	0.06	−2.94	.004*^b^*
Wk 2 $\times$ change in tendon thickness	−14.44	5.88	−2.45	.015*^b^*
Wk 4 $\times$ change in tendon thickness	12.09	7.44	1.63	.11
Wk 8 $\times$ change in tendon thickness	2.17	6.34	0.34	.73
Wk 2 $\times$ change in PSFR	−7.60	9.00	−0.84	.40
Wk 4 $\times$ change in PSFR	2.59	7.13	0.36	.72
Wk 8 $\times$ change in PSFR	−14.71	9.50	−1.55	.12

^*a*^Abbreviation: PSFR = peak spatial frequency radius.

^*b*^Significant variable (*P* < .05).

**Figure 3:**
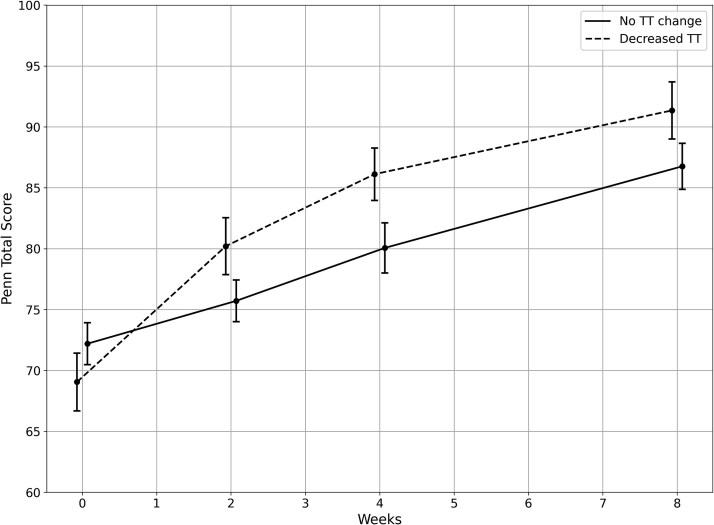
Pennsylvania Shoulder Score Values Over the 8-Week Intervention for Those Who Had a Tendon Thickness Decrease Greater Than the Minimal Detectable Change Versus Those Who Did Not. Error bars represent SEs. Abbreviation: TT = tendon thickness.

**Figure 4:**
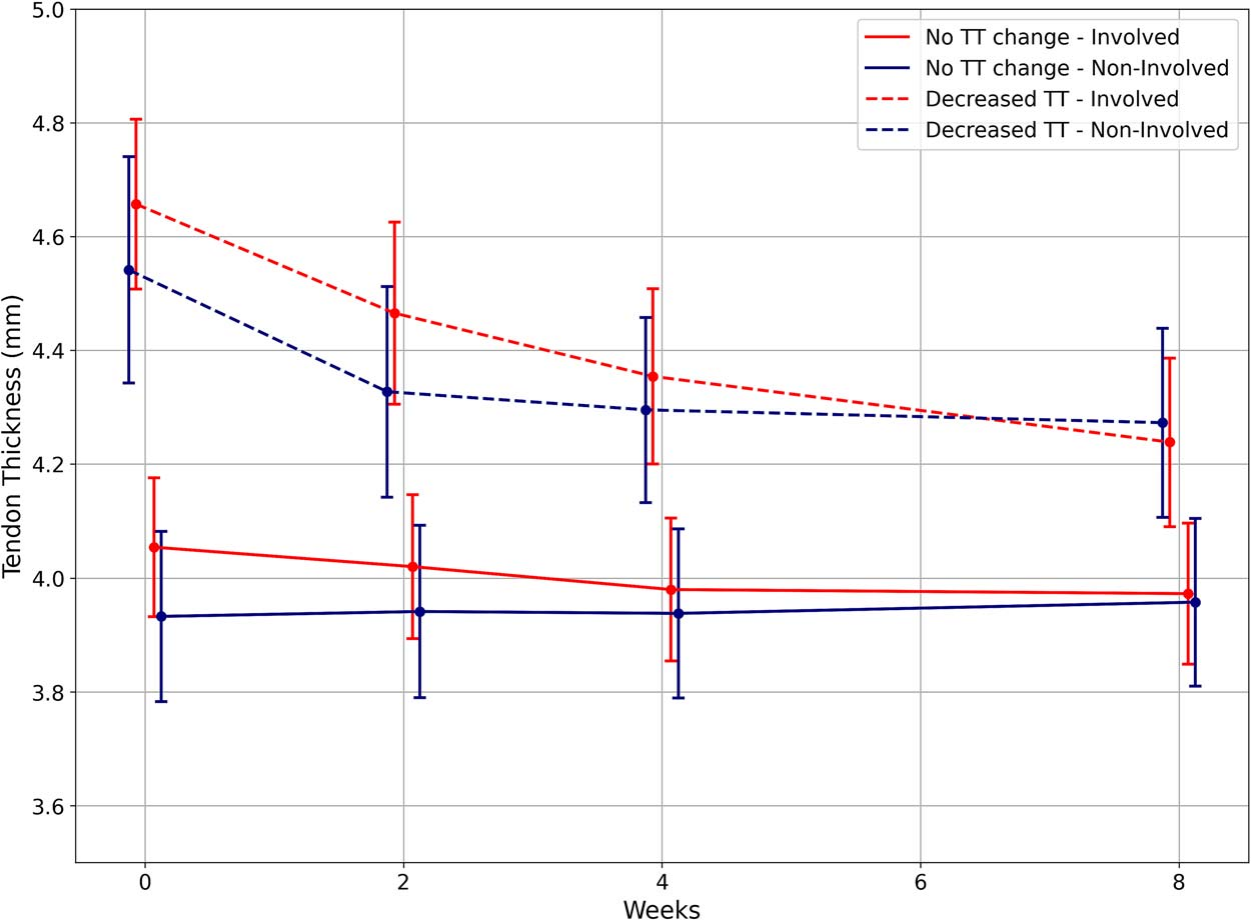
Tendon Thickness Over the 8-Week Intervention for Those Who Had a Tendon Thickness Decrease Greater Than the Minimal Detectable Change Versus Those Who Did Not. Error bars represent SEs. Abbreviation: TT = tendon thickness.

To provide a descriptive visualization of the relationship between changes in Penn Score and tendon characteristics, we plotted the trajectories of individuals who showed a decrease in tendon thickness greater than the MDC (0.3 mm; *n* = 17) and those whose change remained below this threshold (*n* = 30). Figures 3 and 4 present individual and average trajectories in the Penn Score and tendon thickness from baseline to week 2 for these 2 trajectories of observed change. The visual difference in trajectories corresponds to the significant interaction found in the mixed-effects model. There was no significant association between PSFR and Penn Score in the model.

## DISCUSSION

Our study examined the changes in tendon morphology and patient-reported outcomes and the relationship between these changes over an 8-week intervention in patients with rotator cuff tendinopathy. Patient shoulder disability outcomes improved significantly over the 8-week intervention, reaching the minimum clinically important difference at week 8. Concurrently, the average decrease in tendon thickness was statistically significant but smaller than the MDC for this measure. Internal tendon architecture did not change over the 8-week intervention. The significant negative effect of the baseline Penn Score on changes in disability suggests that participants with higher initial disability levels experienced more substantial improvements. This is consistent with extensive literature indicating that individuals with greater initial deficits may respond more to therapeutic interventions.^37^^,^^60–64^

Within the first 2 weeks, the decreased tendon thickness on the involved side was significantly related to improvement in patient-rated shoulder pain and disability. However, this relationship was not sustained over weeks 4 and 8 of the intervention. This early decrease is likely to represent water exudation rather than collagen changes, because of the slow repair rate of the collagen fibers.^65^^,^^66^ This is also further supported by the lack of significant PSFR changes. These findings are similar to those described by Dubé et al,^37^ who found no relationship between changes in tendon thickness and change in disability in their 12-week intervention studies. However, this study used bilateral ratios of tendon thickness, which may reflect changes in both the involved and noninvolved sides, as shown by our data for the noninvolved side. Our results suggest that future studies should be cautious when using ratios or bilateral comparisons. For example, similar changes on both sides can result in an unchanged ratio, which might be mistakenly interpreted as no change in tendon thickness. Therefore, analyzing absolute tendon thickness values of both sides is warranted. It should be noted that in many studies that include exercise as the primary intervention, including the current study, many exercises involve using both upper extremities. This puts load on both shoulders, potentially creating local changes on the noninvolved side. In addition, improvement on the affected side can also lead to performing more bilateral tasks in daily activities that were being avoided in the presence of unilateral pain.

Another discrepancy of our findings with the study of Dubé et al^37^ is that they found 3 subgroups of patients: 1 that did not have a change in the bilateral tendon thickness ratio and 2 that had ratio changes in opposite directions. The majority of our participants showed a decrease in tendon thickness or an unchanged tendon thickness, with very few participants showing increased tendon thickness. The difference in intervention time could partially explain this difference, with longer interventions allowing for healthier hypertrophy (increased collagen fiber count versus increased water content) of the tendon.^67^

The lack of statistically significant findings in the bilateral differences suggests that both the involved and uninvolved sides had comparable baseline tendon thickness and similar changes with the intervention. This points to the possible influence of other factors, such as systemic inflammation. Research has identified that systemic factors, such as metabolic syndrome, can contribute to the development of rotator cuff tendinopathy.^68^ Although conditions related to metabolic syndrome, such as obesity, diabetes, hypertension, and high cholesterol were reported for this study, no measures of proinflammatory cytokines or other objective inflammatory markers were included. Other studies have found increased levels of interleukin 6 and tumor necrosis factor alpha in patients with rotator cuff tendinopathy.^69–71^ Future studies should consider assessing systemic inflammatory markers to explore their potential role in tendon pathology and response to exercise.

### Limitations

This study is not without limitations. The single-cohort design of patients with rotator cuff tendinopathy limits the understanding of the prognostic value of these tendon morphology variables. Given the lack of studies analyzing the relationship between changes in tendon morphology and patient-reported outcomes, this study provides a first step to characterizing this potential mechanism of recovery. However, more longitudinal studies with control groups need to provide proof that tendon structure changes can drive improvement and that they are not just an unrelated consequence of undergoing an exercise intervention, even in the absence of pathology. More interventions should also be studied. Exercise was selected as the only intervention because of its strong supporting evidence.^11^^,^^72–75^ The relationship examined—tendon structure and patient-reported outcomes—may be specific to this intervention and may differ with others. Adjunct treatments were intentionally excluded to avoid confounding influences. Focusing on a single, evidence-based intervention allows for clearer interpretation of these variable relationships within a defined clinical context.

An additional potential limitation is related to the methodology described to characterize internal tendon architecture. This method has shown ability to identify known injured tendon tissue versus healthy tissue,^40^^,^^44^^,^^76^ and is related to collagen organization in tissue histology.^77^ However, The sensitivity of this measure (PSFR) to detect small changes in tendon internal architecture over a relatively short time (8 weeks) is not well established in the literature. It is possible that small changes in the tissue of the tendon happened within our study, but the resultant internal architecture of the tendon did not change enough for our method to be able to capture it.

Further research with larger sample sizes is needed to confirm these findings and understand the underlying mechanisms. Future research should also consider additional factors, such as markers of systemic inflammation, which may influence treatment outcomes in this patient population. Neuromuscular factors, pain and sensorimotor processing and psychosocial factors may also explain the change in patient-reported outcomes as described by Vila-Dieguez et al,^17^ and future research should consider including these to provide a more holistic understanding.

Overall, our findings contribute to understanding the mechanisms underlying patient response to resistive exercise in rotator cuff tendinopathy. By characterizing both tendon size and internal tendon architecture changes and exploring their relationship to disability outcomes, this study provides valuable insights into the complex interplay between tendon structure and clinical outcomes.

## CONCLUSIONS

Changes in tendon thickness were related to improvements in patient-reported outcomes early in the intervention, specifically at 2 weeks. The lack of differences in bilateral tendon structure suggests that both local and systemic inflammatory factors may be at play in tendon recovery. These findings highlight the complexity of recovery in rotator cuff tendinopathy and suggest that further investigation into systemic inflammation, early tendon changes, neuromuscular factors, pain and sensorimotor processing and psychosocial factors could improve treatment outcomes for rotator cuff tendinopathy.

## Supplementary Material

2024_0823_R2_Supplementary_Material_1_conv

2024_0823_R2_Supplementary_Material_2_pzaf_107

## Data Availability

Data are available upon request. All data used for study analyses are available, please contact OVD via email (vilafisio@gmail.com) for further information.
