# Supplementary material for: Relationship Between Tendon Tissue and Shoulder Disability Change During an 8-Week Exercise Intervention for Rotator Cuff Tendinopathy: An Observational Study
Source: Phys Ther. 2025 Aug 29;105(10):pzaf107. doi: 10.1093/ptj/pzaf107 (PMC12494220; doi:10.1093/ptj/pzaf107)
Supplement: 2024_0823_R2_Supplementary_Material_1_conv [file 2024_0823_r2_supplementary_material_1_conv.pdf]

1. Baseline session: receive instruction for level 1 (out of 3) elastic band and number of sets
2. Virtual visits: assess for progression or regression

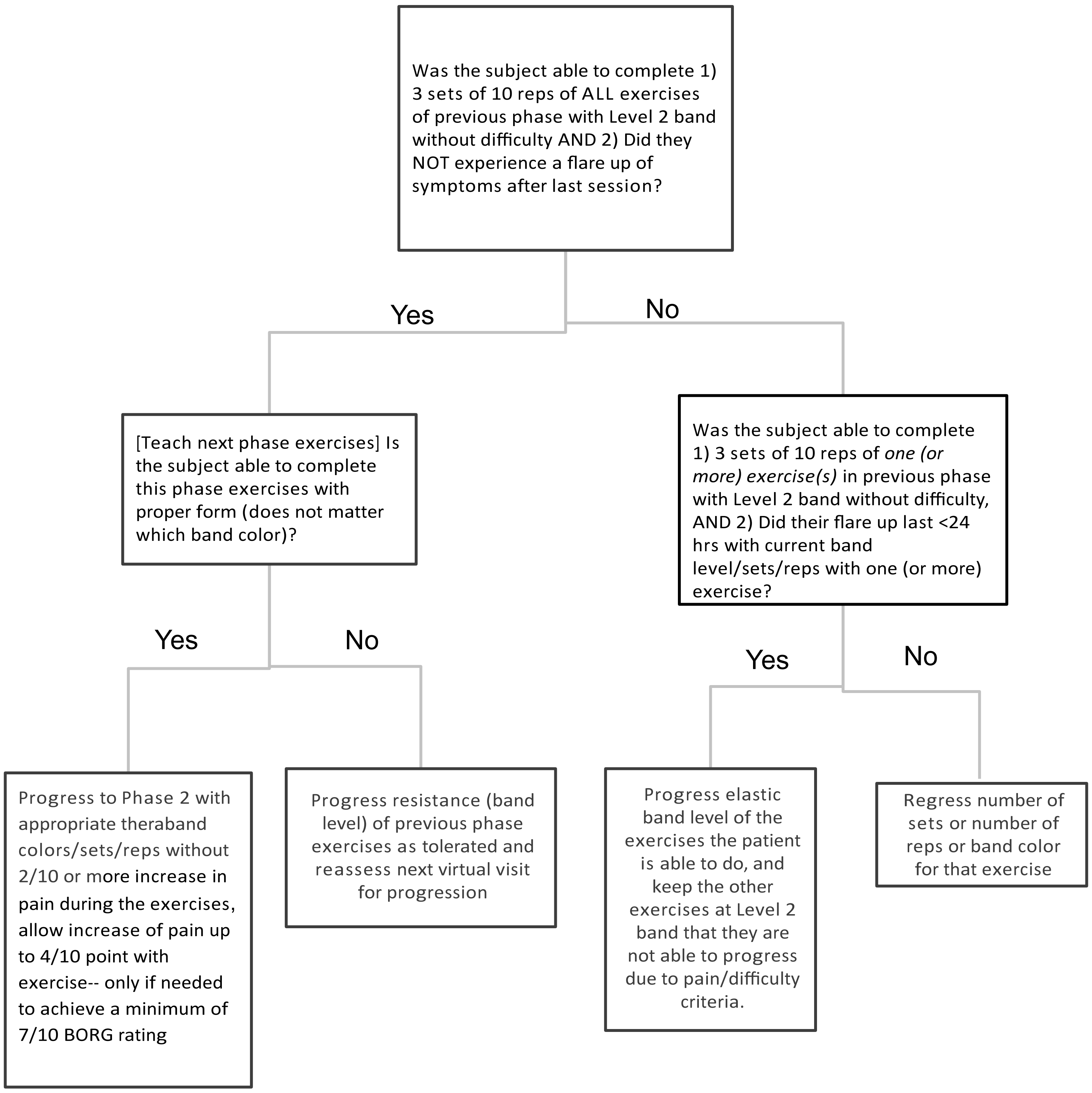

Phase 1

1. Resisted shoulder external rotation (neutral)

• Begin with hand in front of the stomach. Pull away from abdomen, then slowly release. Can use towel in armpit if more comfortable

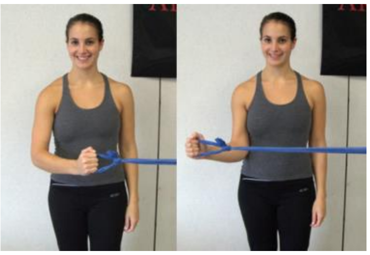

2. Resisted shoulder internal rotation (neutral)

• Begin with forearm out to the side and elbow against body. Pull toward your abdomen, then slowly release. Can use towel in armpit if more comfortable

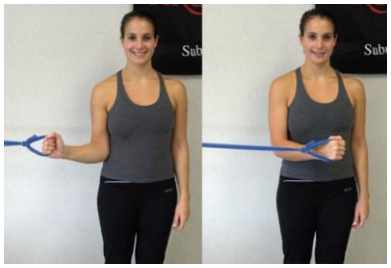

3. Resisted scapular extension

• Begin with arms forward flexed about 45°. Pull band toward you, keeping your elbow bent

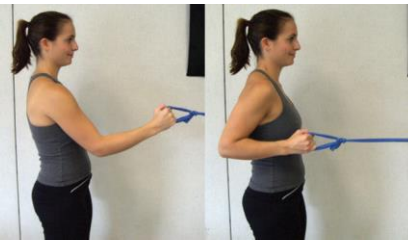

4. Resisted scapular retraction

• Grasp band with both hands and elbows
bent. Pinch your shoulder blades together, which will stretch the band, then slowly release

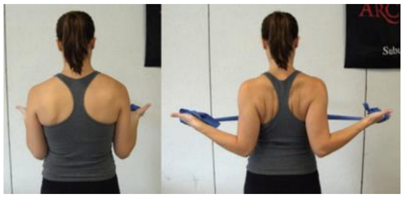

5. Resisted scapular protraction supine

• Grasp band while lying on your back with arm flexed to 90°. Punch arm up toward the ceiling while keeping arm straight. Your shoulder blade should lift off the table

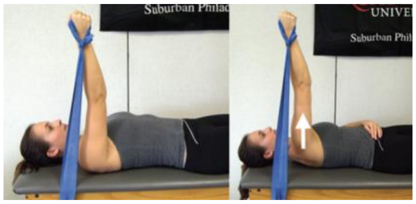

6. Active elevation with upper trap relaxation

•Lift your arm upward while keeping your shoulder relaxed (avoid shrugging). You may use a mirror or your other hand to check to see if your shoulder is lifting up

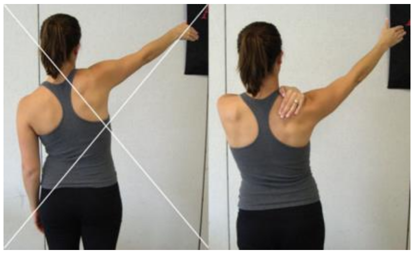

7. Chin tuck with scapular retraction (postural exercise)

• Sitting or standing, tuck your chin and pull shoulder blades down and back. Avoid tilting the head back or looking at the ceiling

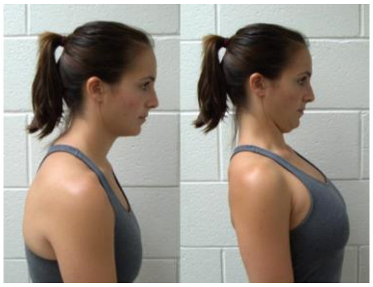

Phase 2

1. Shoulder abduction “scaption” (0°-90°)

• Stand on band and grasp other side, thumb pointing up. Lift band to shoulder level, staying in a plane of movement midway between front and side, then slowly lower

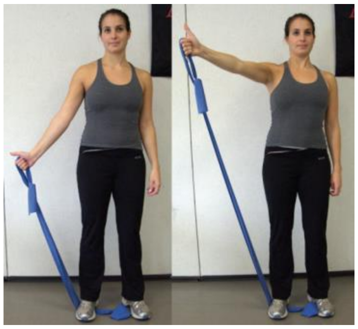

2. Shoulder flexion (0°-90°)

• Stand on band and grasp other side, thumb pointing up. Lift band forward to shoulder level and slowly release

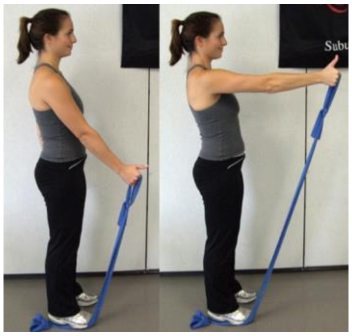

3. Shoulder external rotation with abduction (45°-90°)

• Standing facing doorway, with arm at or below shoulder level and elbow bent 90°. Pull band away from the door, keeping your elbow bent, and slowly release

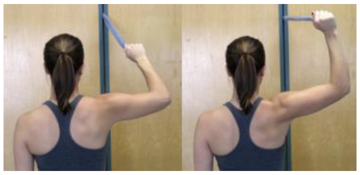

4. Shoulder internal rotation with abduction (45°-90°)

• Stand facing away from the doorway, with arm at or below shoulder level and elbow bent to 90°. Grasp band and pull palms down toward the floor. Slowly release

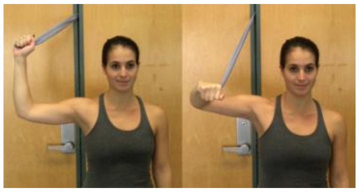

5. Quadruped push-up plus “camel”

• Begin on hands and knees with arms shoulder-width apart. Push downward, causing your upper back to round, then slowly release

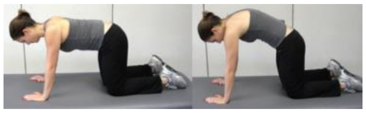

6. Prone shoulder horizontal abduction with scapular retraction “T”

• Turn thumb up and lift arm up toward the ceiling while squeezing shoulder blades toward spine. Slowly lower

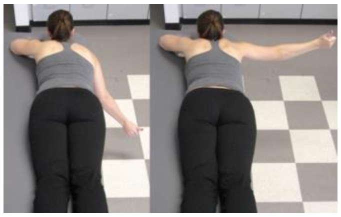

7. Prone scapular retraction and shoulder elevation “Y”

• Turn thumb up and lift arm diagonally above shoulder toward the ceiling while squeezing shoulder blades toward spine. Slowly lower

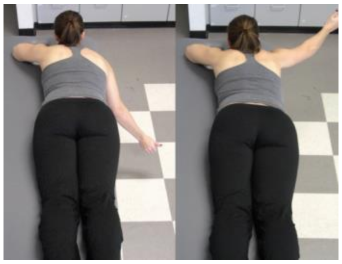

Phase 3 – Continue shoulder abduction, flexion, internal and external rotation from phase 2, and add the following.

1. Push ups on knees

Begin lying on the floor, elbows bent and hands at shoulder width. Lift your body from the ground until the elbows are straight.
Increase difficulty by doing the push up on hands and feet.

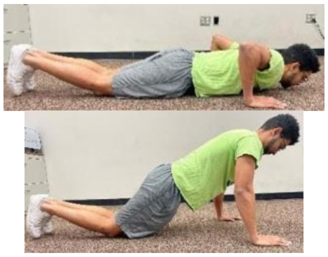

2. Inverted row

Begin lying on the floor, knees bent, hand on a stable desk or a bar, elbows straight. Pull your body up until the shoulders are in line with the elbows.
Increase challenge by keeping your legs straight.

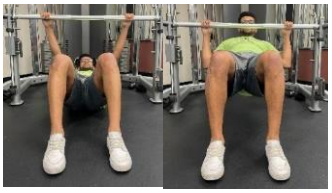

3. Triceps dips

Hands on the edge of a chair, bend your elbows until 90° and then straighten them back until right before being locked.
Increase challenge by keeping your legs straight.

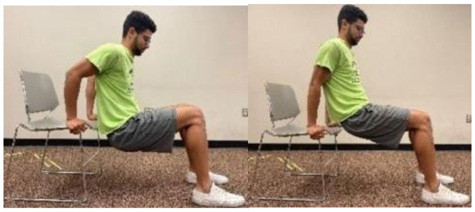

4. Shoulder depression

Between two chairs, place each hand on a chair. With elbows straight, relax the shoulder (passive shrug) and then depress your shoulder as much as possible.
Increase challenge by keeping your legs straight.

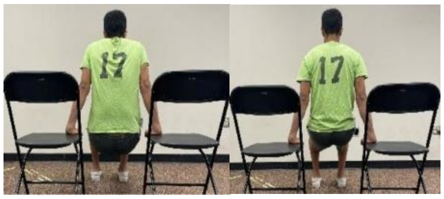

5. Overhead press

Stand on one end of band and grasp the other side. Push the band up until the elbow is fully locked. Slowly return to the starting position.

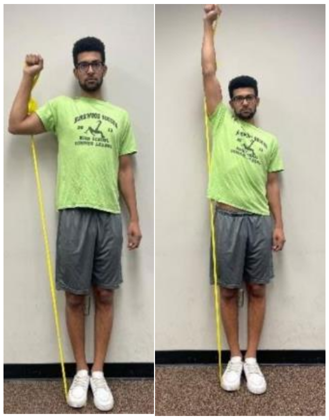

Phase 1 and 2 figures and text were reprinted from Tate et al.[55] with permission from JOSPT.
