# Supplementary material for: Relationship Between Tendon Tissue and Shoulder Disability Change During an 8-Week Exercise Intervention for Rotator Cuff Tendinopathy: An Observational Study
Source: Phys Ther. 2025 Aug 29;105(10):pzaf107. doi: 10.1093/ptj/pzaf107 (PMC12494220; doi:10.1093/ptj/pzaf107)
Supplement: 2024_0823_R2_Supplementary_Material_2_pzaf_107 [file 2024_0823_r2_supplementary_material_2_pzaf_107.pdf]

Did patient-reported outcomes and tendon structure change over the 8-week intervention?

**Variable**	**Estimate**	**Std. Error**	**t-value**	**p-value**
**Table 1.** Estimates for the model with Penn score as the outcome				
(Intercept)	71.06	1.47	48.43	**<0.01**
Week 2	6.28	1.16	5.39	**<0.01**
Week 4	11.19	1.16	9.61	**<0.01**
Week 8	17.36	1.16	14.91	**<0.01**
**Table 2.** Estimates for the model with Tendon Thickness as the outcome				
(Intercept)	4.27	0.10	42.37	**<0.01**
Week 2	-0.09	0.03	-3.58	**<0.01**
Week 4	-0.16	0.03	-6.17	**<0.01**
Week 8	-0.20	0.03	-7.99	**<0.01**
**Table 3.** Estimates for the model with PSFR as the outcome				
(Intercept)	1.58	0.02	79.93	**<0.01**
Week 2	-0.03	0.02	-1.32	0.19
Week 4	-0.01	0.02	-0.64	0.52
Week 8	-0.01	0.02	-0.67	0.51

Were changes in tendon structure associated with change in patient outcomes?

Table 4. Estimates for the model with Penn score change as the outcome (changes from baseline)

**Variable**	**Estimate**	**Std. Error**	**t-value**	**p-value**
(Intercept)	13.87	7.69	1.80	0.08
Week 2	6.12	1.20	5.09	**<0.01**
Week 4	11.60	1.21	9.59	**<0.01**
Week 8	17.36	1.27	13.70	**<0.01**
Baseline Tendon Thickness	1.61	1.77	0.91	0.37
Baseline PSFR	-1.35	5.50	-0.25	0.81
Time Since Onset of Pain	-0.00	0.01	-0.57	0.62
Pain in Dominant vs Non-Dominant	0.30	1.91	0.16	0.88
Baseline Weight	0.03	0.03	1.24	0.22
Baseline Penn Score	-0.27	0.09	-2.97	**<0.01**
Week 2 × Change Tendon Thickness	4.12	4.32	0.95	0.34
Week 4 × Change Tendon Thickness	6.01	4.17	1.44	0.15
Week 8 × Change Tendon Thickness	1.65	3.93	0.42	0.68
Week 2 × Change PSFR	-4.96	5.68	-1.03	0.31
Week 4 × Change PSFR	-1.90	4.49	-0.34	0.73
Week 8 × Change PSFR	-4.78	4.50	-1.06	0.29

Table 5. Estimates for the model with Penn score change as the outcome (Sequential Changes)

(Intercept)	15.26	5.15	2.96	**<0.01**
Week 4	-0.59	1.40	-0.42	0.68
Week 8	0.32	1.38	0.24	0.81
Baseline Tendon Thickness	0.20	1.21	0.17	0.87
Baseline PSFR	-0.12	3.61	-0.03	0.97
Time Since Onset of Pain	-0.00	0.00	-0.50	0.62
Pain in Dominant vs Non-Dominant	0.66	1.27	0.52	0.60
Baseline Weight	0.02	0.02	1.11	0.27
Baseline Penn Score	-0.18	0.06	-2.95	**<0.01**
Week 2 × Change Tendon Thickness	2.34	4.27	0.55	0.58
Week 4 × Change Tendon Thickness	8.11	6.25	1.30	0.20
Week 8 × Change Tendon Thickness	2.93	4.64	0.63	0.53
Week 2 × Change PSFR	-9.44	5.93	-1.59	0.11
Week 4 × Change PSFR	1.20	4.77	0.25	0.80
Week 8 × Change PSFR	-3.56	5.64	-0.63	0.53
